# Soil and Regulated Deficit Irrigation Affect Growth, Yield and Quality of ‘Nero d’Avola’ Grapes in a Semi-Arid Environment

**DOI:** 10.3390/plants10040641

**Published:** 2021-03-28

**Authors:** Maria Gabriella Barbagallo, Giuseppe Vesco, Rosario Di Lorenzo, Riccardo Lo Bianco, Antonino Pisciotta

**Affiliations:** Department of Agricultural, Food and Forest Sciences, Università degli Studi di Palermo, Viale delle Scienze 11, 90128 Palermo, Italy; giuseppevesco79@gmail.com (G.V.); rosario.dilorenzo@unipa.it (R.D.L.); riccardo.lobianco@unipa.it (R.L.B.); antonino.pisciotta@unipa.it (A.P.)

**Keywords:** predawn and stem water potential, grape quality, *Vitis vinifera*, yield

## Abstract

The present work studied the effect of two consecutive years of regulated deficit irrigation (RDI) compared to rain fed management on the vegetative growth, yield, and quality of ‘Nero d’Avola’ grapes. The trial was conducted separately in two soils (vertisol and entisol) located at the top and bottom hillside of the same vineyard. Vertisol was characterized by greater depth, organic matter, exchangeable K_2_O, and total N than entisol. RDI was based on an irrigation volume at 25% of estimated crop evapotranspiration (ET_c_) up to end of veraison and 10% of estimated ET_c_ up to 15 days before harvest. Predawn water potential (PDWP) was used as indicator of plant water status and irrigation timing. No difference in irrigation management was evident between vertisol and entisol. Under Mediterranean climate conditions, RDI was able to enhance grape yield and vegetative growth, especially in vertisol, but it reduced berry titratable acidity and total anthocyanins. ‘Nero d’Avola’ showed to adapt to drought conditions in the open field. Both soil type and irrigation regimes may provide opportunities to obtain different ‘Nero d’Avola’ wine quality and boost typicality.

## 1. Introduction

In recent years, Mediterranean regions are being affected by marked climate changes, primarily characterized by reduced precipitation, greater concurrence of temperature extremes and drought during the growing season, and increased inter-annual variability in temperatures and rainfall [1,2]. For this reason, the wine regions of southern Europe will experience, or are already experiencing, a modification of their traditional terroirs, which in turn, will significantly increase the variability of yields and wine quality attributes, style, and typicality [3].

Soil is an important part of terroir, but great wines are not related to one soil type [4]. Great wines are produced worldwide on a wide variety of soils and it is not possible to define a high-quality potential vineyard soil in terms of soil texture, soil type, or soil minerals [5]. Soil depth, soil water holding capacity, and soil nitrogen level are important characteristics influencing vine behavior in terms of vigor, quantity, and quality of grapes. In Bordeaux (France), the best soils are considered those characterized by free draining, lack of water logging in the rooting zone, and limited water availability during ripening [6]. Indeed, some researchers give more importance to soil physical properties determining water supply to the vine rather than to soil chemical constituents [7]. Whether soil plays a primary and direct role on wine quality or it indirectly determines wine quality, through its effects on vine growth, canopy density, and vigor must be still established [8].

Soil texture influences soil water holding capacity and consequently the progressive water release for vine root uptake. Regular but limited water supply before veraison reduces shoot and berry growth and increases berry anthocyanin and tannin concentration [4]. van Leeuwen et al. [9] reported that different non-irrigated cultivars grown on clay soil displayed a higher level of sugar and lower total acidity compared to those on gravel and sandy soils. However, site and vintage seem to play major roles (rather than soil texture) on vine size and wine sensory characteristics [8].

Generally, high-quality red wines need moderate water deficit and mineral supply, especially nitrogen [4]. Hence, irrigation may be needed to avoid severe vine water stress occurring in some vintages and in soils characterized by low holding capacity. In recent times, irrigation is becoming a common practice also to prevent the negative effects of climate change [10,11] even in those areas where grapevines are traditionally rain-fed. Indeed, irrigation allows to standardize yield and quality of grapes over the years, especially when rainfall is too low and, in some circumstances, certain irrigation techniques allow for significant water savings [3].

Nevertheless, water-deficit-induced different effects on grapevine yield and quality components depend on timing, intensity, and duration of plant water stress [12]. The adoption of regulated deficit irrigation (RDI) in the vineyard requires setting irrigation timing and volume to keep the vines within the stress level ranges identified for the different phenological phases. Generally, moderate-to-high water stress imposed with RDI positively influenced the amount of berry secondary metabolite, including total anthocyanin content [13,14], and induced a reduction in berry size [15,16,17]. However, in the areas with rainy spring and/or high soil water reserves, irrigation may be used to modulate vine water stress levels only in mid to late summer [18].

In deficit irrigation strategies, it is useful to define irrigation volume based on fractions of crop evapotranspiration (ET_c_) when crop coefficients are well adjusted and the reference evapotranspiration (ET_0_) is available [19]. This, however, may create some indecision as plant water stress development depends not only on the fraction of water consumption replaced in the soil, but also on soil water holding capacity, on growing conditions, and so on [20]. Different methods to evaluate the vine’s water status have been implemented and are available, with the advantage of possible automation [21,22]. One alternative approach is the use of plant water potential as an irrigation signal. Predawn and stem leaf water potentials are the most-used methods for the current irrigation management of vineyards. Although they are considered good indicators of vine water status [23], not all researchers consider them equally correlated with soil water content [24]. Predawn leaf water potential, in particular, is thought to estimate soil moisture better than the other methods [23,25,26] because it is nearly not affected by environmental factors, representing a situation of stable equilibrium between soil and atmosphere. On the contrary, other authors [27,28] showed that midday stem water potential is a very accurate measure and a better indicator of grapevine water status than predawn leaf water potential for the irrigation management of grapevines. These different results may be in part explained by the fact that grapevine cultivars withstand different levels of drought as they can show similar values of stem water potential, but at the same time, different values of predawn water potential [29]. For this reason, it is difficult to establish a range of stem water potential to manage vineyard irrigation that would be valid for all varieties. Hence, in this experiment, we decided to use predawn leaf water potential to establish irrigation timing.

Nero d’Avola (also known as Calabrese) is characterized by high vigor and yield [30] and is the main black cultivar in Sicily, accounting for about 15,500 ha (15.6% of total Sicilian vineyards) [31]. ‘Nero d’Avola’ is in 17 Sicilian PDO wines with a production of 31 mL out of 150 mL of total Sicilian PDO wines [32]. Recently, ‘Nero d’Avola’ has been planted in other parts of the world including Australia, California, and South Africa, also thanks to its ability to withstand dry, hot conditions. In one of the few studies carried out on ‘Nero d’Avola’, the vines showed a rather isohydric behavior, its degree varying with potassium availability when vines were subjected to moderate drought stress [33]. Nevertheless, the real ability of ‘Nero d’Avola’ to adapt to drought conditions in open field must be still determined.

The trial was carried out in Sicily, during two vegetative seasons (2005 and 2006) and in a ‘Nero d’Avola’ vineyard characterized by two soils different for their position in the vineyard (one at the top, the other one at the bottom of a slope), depth, water holding capacity, organic matter, and nitrogen content. The objectives of the present study were to test (1) the effect of soil and RDI irrigation on vigor, yield, and quality of ‘Nero d’Avola’ grapes; and (2) the ability of ‘Nero d’Avola’ vines to adapt to drought conditions in open field.

## 2. Materials and Methods

### 2.1. Experimental Site

The experimental site was located within the Alcamo D.O.C. area, in the hinterland of western Sicily (37°55′11.66″ N; 13°04′10.03″ E), 300 m a.s.l. The trial was carried out for two years (2005–2006) in a drip-irrigated vineyard with eight-year-old ‘Nero d’Avola’ vines grafted onto 1103 P rootstock. The rows were spaced 2.40 m apart, with 0.95 m between vines on the row. Vines were trained to a vertical shoot positioned trellis (VSP) and pruned to two buds per spur, spaced at approximately 15 cm in a single cordon (Table S1). Climate trends of the two years of study are perfectly in line with climate trends of recent years (2015–2019, data not shown); therefore, data collected in 2005 and 2006 can be considered relevant and representative of the present climatic conditions at the experimental site.

The irrigation system used 4 L h^−1^ pressure-compensating inline emitters spaced 0.95 m apart. Drip-irrigation lines were placed ~40 cm above ground. The amount of water applied in each irrigation treatment was measured using flow meters.

Soil management practices consisted of growing a cover crop (*Vicia faba*) during winter and burying its biomass in the soil in April. Three shallow tillage events (10–12 cm deep), from spring to summer, were carried out to control weeds, prevent crust formation and ultimately reduce soil evaporation.

Weather data were collected with an automated weather station located at 680 m of distance (37°45′50.61″ N, 13°04′13.73″ E) from the experimental vineyard.

### 2.2. Soil Characteristics

Analysis of 20 profiles allowed for the individuation of two distinct types of soil, one at the top, the other one at the bottom of the hill (slope 10%). The two clay soils were classified using the soil taxonomy [34] as Typic Xerorthents (entisol) at the top of the hill, and Chromic Haploxererts (vertisol) at the bottom of the hill. The entisol was characterized by a loamy texture and clay-montmorillonite with a low amount of skeleton and pH of 7.8. The entisol profile was Ap-C and its depth was up to 50–60 cm. Organic matter (0.98%), total salinity (0.98 mS/cm), CaCO3 (5.4%) and total N content (658 ppm) were low, while exchangeable K_2_O was high (294 ppm).

The texture of vertisol was characterized by 41.3% of clay; the profile was very deep (>100 cm) and the amount of skeleton was generally low. The pH was 7.6 and the organic matter was high (2.03%). Total salinity (1.07 mS/cm) and CaCO3 content (5.1%) were low. Exchangeable K_2_O was high (402 ppm), while total N was medium/low (966 ppm). When not cultivated during the dry season, both soils exhibited deep cracks (to a depth of 80–100 cm in the vertisol) (Table S1).

### 2.3. Irrigation Treatments

In 2005 and 2006, the following treatments were applied:

(1) rain-fed—vines grown without irrigation water;

(2) regulated deficit irrigation (RDI)—vines irrigated at 25% of estimated ET_c_ up to the end of veraison and at 10% of estimated ET_c_ up to 10 days before harvest. Predawn leaf water potential (PDWP) was used to establish irrigation timing according to specific thresholds differing by phenological stages. In particular, the irrigation was applied when PDWP reached values below –0.5 MPa until the end of veraison, and below –0.7 MPa after the end of veraison until 10 days before harvest (Table S1). ET_c_ was calculated with the method proposed by Allen et al. [19] using the Penman–Monteith equation to calculate reference evapotranspiration (ET_0_) and tabulated crop coefficients (Kc). The rain-fed treatment was considered the reference (control) as this is the common irrigation management in the area, and a full irrigated reference (irrigation = 100% ET_c_) would be meaningless for the production of quality wines.

### 2.4. Experimental Design

The experiment layout was a split-plot design with the two soils as the main plots and the two irrigation treatments as sub-plots. The experimental plot was first divided in six main-plots of six rows each for the assignment of the entisol and vertisol. Each main-plot was further divided in two sub-plots for the assignment of the two irrigation treatments. The elemental plot comprised three adjacent rows (two buffer rows and a central one for data collection) and was replicated three times. Ten vines with similar vigor, measured at the beginning of the trial, for each replicate were considered for data collection (Figure S1).

### 2.5. Soil Water Content

Soil water content was monitored with one Diviner 2000 capacitance probe (Sentek Environmental Technologies, Stepney, South Australia) per treatment and soil, each placed in access tubes installed to a depth of 1 m and located in the row line at 47 cm from the trunk in all treatments. Moreover, field capacity and wilting point were determined using Richard’s plates [35] to calibrate the probe with the vineyard’s soil type. Data from the probes were expressed as a percentage of volumetric water content. Measurements were carried out every 10 days starting about 1 month after fruit set. For each, sampling date, treatment and soil, and soil water content measurements (six) were taken at 15 cm to 100 cm depths (at about each 15 cm).

### 2.6. Ecophysilogical Measurements

The measurements of PDWP were taken before sunrise, with stem water potential (SWP) measurements taken at midday (between 12:30 to 13:30 h), using a Scholander pressure chamber [36] and selecting four leaves, from four different vines and opposite to clusters for each treatment and replication. Following the methodology described by Williams and Araujo [23], SWP was measured on mature leaves with similar age in the shaded canopy side of the vertical trellis. At least 60 min before, the leaves were enclosed in plastic bags and covered with aluminum foil [23]. Four measurements per replication of PDWP and SWP were collected starting at fruit set every 10 days in each year.

Stomatal conductance (g_s_) (four per replication) was measured at midday in the same days and on similar leaves (but exposed to the sun) as those of water potentials, using an AP4 porometer (Delta-T Devices Ltd., 130 Low Road, Burwell, Cambridge, UK).

### 2.7. Leaf Area and Pruning Mass

At two phenological stages (pea size and harvest), 10 shoots per treatment and replication were collected, and total leaf area (TLA) of primary and lateral shoots was measured using an LI-3100C area meter (Li-COR Environmental, 4647 Superior Street Lincoln, NE, USA). During winter of both years, pruning wood of ten vines for each replication and treatment was weighed and cane number per vine was counted.

### 2.8. Yield and Grape Composition

At full maturity, the clusters of 10 vines per treatment and replicate were harvested, counted and weighed and then used to calculate yield per vine and average cluster weight. For each replication and treatment, 100 berries were randomly collected and weighed, and average berry weight was calculated. Total anthocyanins (expressed as mg kg^−1^ of grapes and mg/berry) [37] were measured from a sample of 25 berries per replication and treatment. The berries were randomly collected from different sections of the bunch (top, middle, bottom, inner and outer portions). The skins of each sample were separated from the pulp and placed in a flask containing 25 mL of tartaric buffer (500 mL of distilled water, 5 g of tartaric acid, 22 mL of 1 N NaOH, 2 g of sodium metabisulphite and 120 mL of 95% ethanol; pH 3.2). The buffer volume was adjusted to 1 L with distilled water. Skins were placed in the buffer for 4 h at room temperature prior to homogenization and centrifugation. The supernatant was collected in a 100 mL volumetric flask. The residue was washed again with tartaric buffer, added to the volumetric flask, and the volume was raised to 100 mL with the same buffer. The extract (10 mL) was diluted 25 times with acidified ethanol (ethanol, H2O and concentrated HCl, 70:30:1 *v/v/v*), and the absorbance was read at 540 nm using a UV–vis spectrophotometer (Varian Cary 50 Bio UV–vis Spectrophotometer, McKinley Scientific, Sparta, NJ, USA).

For each replication and treatment, bunches were hand pressed, and the juice was recovered to measure total soluble solids (TSS, °Brix) using an Atago PR-32 digital refractometer (Atago, Tokyo, Japan) and titratable acidity (TA) using a Crison Compact Titrator (Crison Instruments, Barcelona, Spain) by raising pH to 7 with 0.1 N NaOH (expressed in g L^−1^ of tartaric acid).

### 2.9. Statistical Analysis

Productive, qualitative, vegetative, and soil water content data were processed by a two-way mixed model analysis of variance (ANOVA) to evaluate the effects of the main factors (soil and irrigation) and their possible interaction. Year was considered as a random factor.

In each season, PDWP and SWP data were processed by repeated measures two-way ANOVA (main factors: soil and irrigation). Then for each date, Tukey’s multiple comparison test was used to detect different means. Moreover, in each season, the relationships between predawn water potential (PDWP) and stem water potential (SWP) and between these parameters and stomatal conductance (g_s_) were tested using regression analysis. Difference between slopes were tested by the Student’s *t*-test. Log-transformation of data was performed, when necessary.

All statistical procedures were performed using R (R Core Team, 2020) [38]. Mixed model ANOVA was performed using the ‘lme4’ R library [39].

## 3. Results and Discussion

### 3.1. Meteorological Conditions, Soil Water Content and Ecophysilogical Measurements

Meteorological conditions were typical of Mediterranean summers; the mean of maximum temperatures ranged from 26 °C to 31 °C between June and September over the two years. Total ET_0_ from April to September slightly differed between 2005 and 2006 with 758 mm and 830 mm, respectively (Table 1).

The summer of 2005 was warmer (except for August) and less rainy (except for June) than the summer of 2006 (Table 1). In the cooler months (October-April), there was about 43% more rain in 2005 (621.4 mm) than in 2006 (355.4 mm). Conversely, the same period October-April was warmer in 2006 than in 2005, except for March.

**Figure 1:**
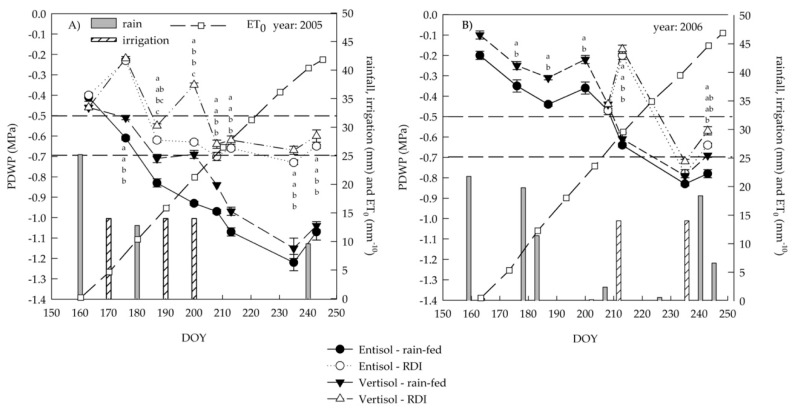
Seasonal variation (DOY, day of the year) in predawn leaf water potential (PDWP) of ‘Nero d’Avola’ grapevines grown in two soils (entisol and vertisol) under rain-fed and regulated deficit irrigation (RDI) treatments in 2005 (**A**) and 2006 (**B**). Bars show rainfall and irrigation events. Cumulative ET_0_ is shown from pea size phenological stage (DOY 160) to ten days before harvest (DOY 245). In 2006, the irrigation started at the end of July (DOY 206). Error bars indicate standard errors (*n* = 12). Dashed horizontal lines indicate the PDWP threshold values for irrigation. When present, different letters indicate significant differences among treatments for a specific date (Tukey’s multiple range test, *p* < 0.05).

In 2005, three irrigation events were carried out: one in the first week of July (DOY 173), the second at the beginning of veraison (DOY 191) and the third at the end of veraison (DOY 204). In 2006, the irrigation started at the end of July (DOY 206), just before veraison, a month later than in 2005, because there was a rainfall event of 31.2 mm at the beginning of July (from DOY 177 to DOY 183) (Figure 1), which prevented grapevine water stress. The last irrigation was in the second half of August (DOY 234). The total irrigation volume was 42 mm in 2005 and only 28 mm in 2006, regardless of soil type, as the established PDWP threshold was reached three and two times during the season in 2005 and 2006, respectively (Figure 1).

**Figure 2:**
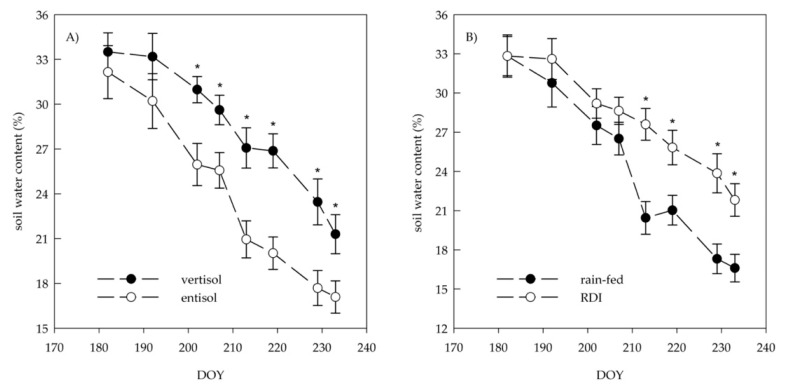
Variation of soil water content (%) over the season in two soils (vertisol and entisol) (**A**) and irrigation treatments (regulated deficit irrigation (RDI), and rain-fed) (**B**). Error bars indicate standard errors (*n* = 24); DOY, day of the year. When present, asterisks indicate significant differences between means (Tukey’s test, *p* < 0.05).

The amount of soil water content varied according to soil characteristics and irrigation treatment (Figure 2A,B). Vertisol was characterized by a lower reduction in the percentage of water content than entisol during summer (Figure 2A). As expected, water depletion occurred more rapidly in rain-fed than irrigated soils, showing significant difference since veraison (DOY 213) (Figure 2B). In detail, vertisol and RDI displayed the same water content pattern as entisol and rain-fed condition (Figure 2A,B).

Although differences in vine water status were expected in relation to soil type, in either vintage, the two soils induced PDWP differences that, although statistically significant in almost all the dates, were not such as to modify RDI management during the season (Figure 1). This may be because the vines grown in vertisol, which was characterized by a deeper profile, exhibited a higher biomass with more root development [40]. Indeed, previous studies showed that marked differences in soil texture, such as between sandy, gravelly and clayey soils, are needed to show soil-dependent responses of stomatal sensitivity to PDWP [41].

However, after the first irrigation of 2005, the PDWP increased more in vertisol than entisol where it remained unchanged. In 2005, PDWP reached very low values (−1.3 MPa) in the rain-fed vines (Figure 1A), which experienced high and prolonged water stress. For the weather conditions of summer 2006, the vines presented less leaf water stress and the values of PDWP did not go below −0.9 MPa (Figure 1B).

**Figure 3:**
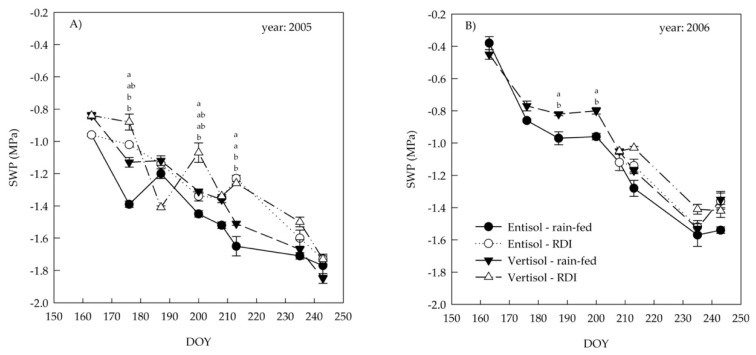
Variation in stem water potential (SWP) of ‘Nero d’Avola’ grapevines grown in two soils (entisol and vertisol) under rain-fed and regulated deficit irrigation (RDI) treatments in 2005 (**A**) and 2006 (**B**). In 2006, the irrigation started at the end of July (DOY 206). Error bars indicate standard errors (*n* = 12). When present, different letters indicate significant differences among treatments for a specific date (Tukey’s multiple range test, *p* < 0.05).

A progressive decrease in PDWP and SWP was evident during the season, even in the irrigated treatment (Figure 1 and Figure 3). At DOY 176, 200 and 213 of 2005, SWP was significantly higher in irrigated than rain fed vines (Figure 3A).

In 2006, before the beginning of the irrigation (DOY 187 and 200), vertisol showed significantly higher SWP values than entisol. After the irrigation began (DOY 208), the SWP differences among treatments were less evident (Figure 3B).

In both years, in the last two dates, close to harvest, the SWP values did not show any difference among soils and irrigation treatments (Figure 3A,B).

Slopes of the relationship between PDWP and SWP did not significantly differ between soil types (Table 2). On this basis, soil data were pooled and recalculated statistics are reported in Table 3. Linear regressions between PDWP and SWP were obtained between years and irrigation regimes, as found by Carbonneau et al. [25], while others found an exponential function [29,42]. The variations in regression slopes observed were probably due to the differences in water stress level of the rain-fed treatment, in the water amount supplied through irrigation, and in the environmental factors between the two years.

Under rain-fed conditions, r^2^ values were high (Table 3), and the slopes detected in the two years were significantly different, such as those obtained under RDI treatments (Table 4).

**Figure 4:**
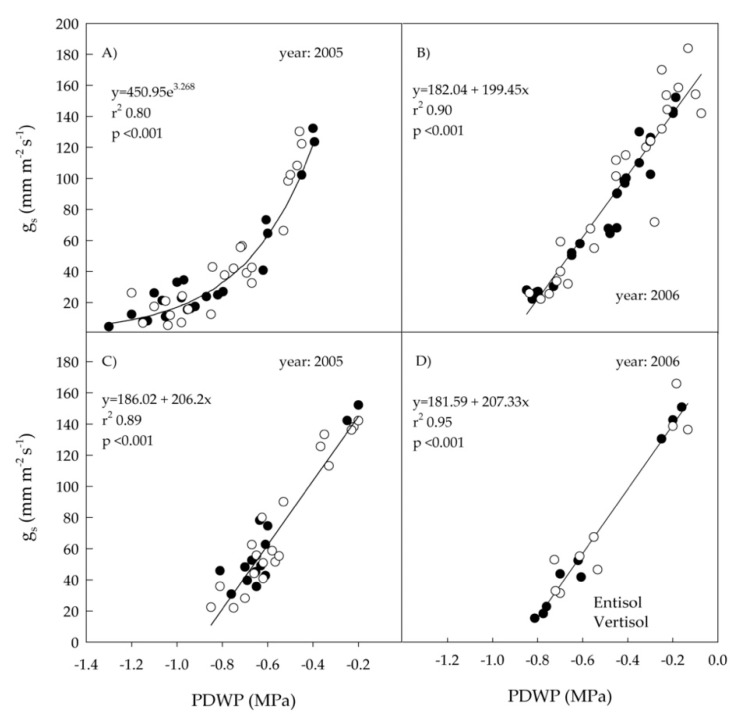
Relationships between stomatal conductance (g_s_) and predawn leaf water potential (PDWP) of ‘Nero d’Avola’ vines grown in two soils (entisol and vertisol) under rain-fed conditions in 2005 (**A**) and 2006 (**B**), and under regulated deficit irrigation (RDI) in 2005 (**C**) and 2006 (**D**). Each point is the mean of four measurements. Data from the two soil types were similar (*t*-test of coefficients, *p* < 0.05) and pooled together for each year and irrigation treatment.

**Figure 5:**
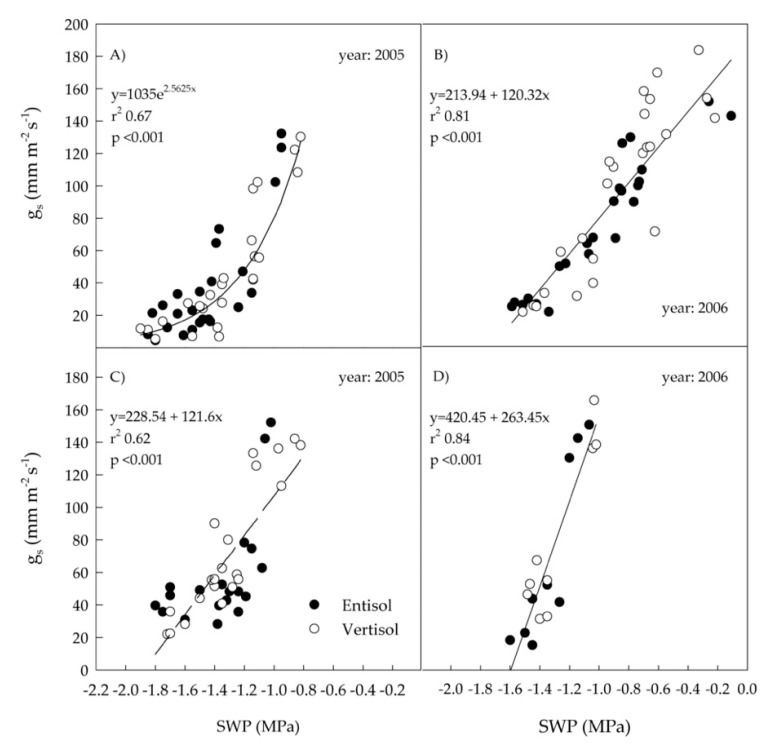
Relationships between stem water potential (SWP) and stomatal conductance (g_s_) of ‘Nero d’Avola’ vines grown in two soils (entisol and vertisol) under rain-fed conditions in 2005 (**A**) and 2006 (**B**), and under regulated deficit irrigation (RDI) in 2005 (**C**) and 2006 (**D**). Each point is the mean of four measurements. Data from the two soil types were similar (*t*-test of coefficients, *p* < 0.05) and pooled together for each year and irrigation treatment.

Comparing the two irrigation strategies in both years, the slopes of the relationships between PDWP and SWP were similar for RDI and rain-fed treatments in 2005, while they were significantly different in 2006 (Table 4). Indeed, in 2006 and rain-fed condition, the slope was almost twice as high as that found in RDI (Table 3 and Table 4). The SWP values of the RDI treatment were less variable in 2006 (from −1.04 to −1.6 MPa) than in 2005 (from −0.8 to −1.78 MPa), probably because in this year, the irrigation began later (at veraison in 2006, at early berry growth in 2005) and the old leaves sampled were less reactive. Also, the slopes of the relationships between g_s_ and PDWP and g_s_ and SWP did not significantly differ between soil types. On this basis, soil data were pooled and recalculated statistics are reported in Figure 4 and Figure 5.

A good linear relationship between g_s_ and PDWP was found in the RDI treatment in 2005 and in both treatments in 2006 (Figure 4B–D), while an exponential relationship was obtained in the rain-fed treatment of 2005 (Figure 4A). Similarly, low PDWP values of −0.8 MPa up to −1.3 Mpa (severe stress) as those measured in our rain-fed conditions of 2005 induced a drop of g_s_ below 50 mmol H_2_O m^–2^ s^–1^ and the relationship between g_s_ and PDWP became exponential, while in the other situations the relationship was always linear [29,43]. A linear relationship was also detected in both years between g_s_ and SWP (Figure 5B–D) except in the rain-fed treatment of 2005 where an exponential relationship was observed (Figure 5A), with r^2^ values generally lower in 2005 than in 2006. In 2006, the slope of the line was higher in the RDI than in the rain-fed treatment (Figure 5B,D).

At relatively high values of PDWP, a wide range of SWP and g_s_ values have been reported, demonstrating also the interaction between these parameters and other environmental factors such as VPD and air temperature [26].

### 3.2. Leaf Area and Cane Mass

There was a significant effect of the soil on total leaf area per shoot (TLA) and cane weight at pruning, while the irrigation treatment influenced only total leaf area per shoot (TLA). In particular, vertisol and RDI irrigation showed the highest TLA at harvest (Table 5). This is in agreement with previous studies where vines grown in vertisol, located at the bottom of the slope, exhibited higher biomass in terms of yield, surface area, and pruning wood [44], together with more efficiency and/or root development [40] because this soil showed a higher depth and water availability [45], but also higher organic matter, total N and K_2_O exchangeable. For sites on hillsides, erosion plays a major role for soil depth and fertility [44,46].

**Figure 6:**
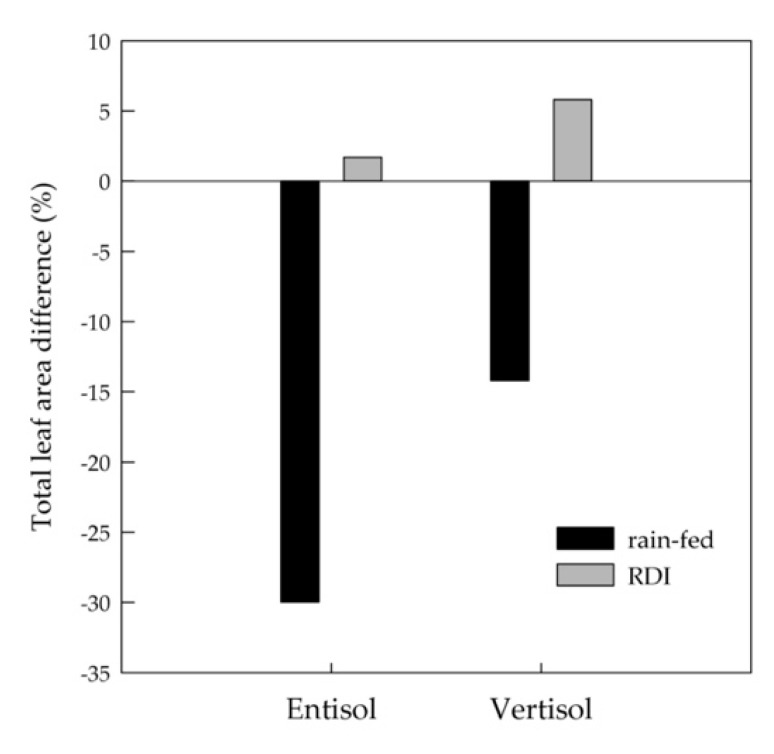
Difference in total leaf area from pea size to harvest (average of two years) for ‘Nero d’Avola’ vines grown in two soils (entisol and vertisol) and under rain-fed and regulated deficit irrigation (RDI) treatments.

The difference in total leaf area between the two irrigation treatments was mostly due to a higher percentage of leaf abscission in the rain-fed vines, mainly in entisol (Figure 6). The leaf fall was especially from the main shoots, while the leaf area of laterals in RDI vines did not change or slightly increased (data not shown). Vertisol and RDI treatment were responsible for increasing of vegetative parameters, since they kept vegetative sinks functioning during ripening, thus diminishing the percentage of leaf shedding and leaf senescence after veraison compared to entisol and the rain-fed treatment. A reduction of secondary growth and leaf area abscission after veraison [47,48,49,50,51], especially in terms of the basal leaves of the main shoots and most of non-lignified lateral shoots [52], are the most noticeable effect of arid conditions and extreme drought, but they vary depending on the meteorological conditions of the vintage. Under drought conditions, leaf abscission is encouraged by petiole vulnerability to cavitation [53]. Basal leaf shedding is thought as an adaptation mechanism to drought conditions by reducing the transpiration process [54].

### 3.3. Yield and Grape Composition

As for productive parameters, yield, cluster, and berry weight changed according to the differences of soil water content and irrigation management. Indeed, the effects of soil and irrigation were significant on yield per vine, cluster, and berry weight (Table 5). Yield per vine was higher in vertisol than in entisol and the difference was due to a higher cluster and berry weight. Moreover, vertisol had a higher influence on yield rather than RDI treatment (Table 5). The interaction between soil and irrigation was never significant for the productive parameters (Table 5). Reductions of berry weight under rain-fed conditions have been already reported [55,56,57,58,59,60] and are most likely due to a reduction of cell expansion [56]. Soil type and irrigation treatment did not affect TSS content, while they had a significant influence on TA (Table 6). RDI reduced TA and anthocyanin concentration (mg kg^−1^), while entisol induced a higher anthocyanin concentration than vertisol. The interaction between soil and irrigation was never significant for quality parameters (Table 6).

Similarly, entisol, located in the higher part of the hill, induced a higher quality of grapes, especially for total anthocyanins, while deep and fertile vertisol, placed at the bottom of the slope, produced a lower quality of grapes [4], especially under irrigated management. Neither soil nor irrigation influenced the must sugar. However, in the literature, contradictory results regarding the influence of irrigation on yield and must sugar have been obtained depending on vintage, cultivars and rootstocks, time and intensity of water deficit [13,14,59,60,61,62,63,64,65], as well as water amounts distributed during the season [57], in the ripening period, and near harvest.

A decrease of titratable acidity in irrigated vines has been previously found [66] as in this study, and this is most likely due to a higher availability and uptake of potassium from the soil. A higher presence of potassium in the berries of irrigated vines may induce a higher salification of must acids [67,68,69,70]. Moreover, the increase in berry size of irrigated vines could cause a dilution effect on titratable acidity.

The expression of anthocyanin biosynthetic genes is affected by seasonal water availability throughout the progress of ripening [13,14,71], and moderate-strong water stress during this period increased anthocyanin synthesis [14,72]. In this study, the higher berry weight obtained in vertisol and RDI irrigation led to a lower skin anthocyanin level; this is due to a reduction a berry number per kg of grapes [17] regardless of the effect on anthocyanin synthesis. Generally, the reduction of berry size leads to an increase of total secondary metabolites and a higher grape quality potential for red wine-making [9].

## 4. Conclusions

Irrigation could mitigate the negative effect of the warm climate and rainfall scarcity. Indeed, in the climatic condition of the study, the irrigation was able to enhance yield and vegetative growth, especially in vertisol, without any influence on sugar but with a decline of titratable acidity and total anthocyanins. Irrigation scheduling and volumes using PDWP thresholds was similar for entisol and vertisol. The ‘Nero d’Avola’ vines showed a fairly good ability to adapt to drought conditions by dropping basal leaves.

Different grape yield and quality in the two soils suggests harvesting grapes from the two soils at the same time but keeping them separate; or delaying harvesting time in the vertisol to enhance wine quality and/or distinguish the types of wines achievable. Further investigation is needed to understand how different wines from grapes of the two soils can be and if unique traits and flavors can be obtained to promote typicality.

## Figures and Tables

**Table 1:** Long term monthly average of temperature (T), rainfall and estimated ET_0_ located at 680 m of distance (37°45′50.61″ N, 13°04′13.73″ E) from the experimental vineyard.

Year	Month	Jan	Feb	Mar	Apr	May	Jun	Jul	Aug	Sep	Oct	Nov	Dec
	T min (°C)	4.80	3.42	7.07	8.78	13.81	17.24	20.30	18.68	17.18	13.99	9.64	6.09
2005	T max (°C)	9.76	9.10	15.10	16.33	24.28	27.93	31.33	29.88	27.07	22.93	17.51	11.97
	rainfall (mm)	88.8	77.2	55.20	121.8	13.4	40.2	12.80	16.00	29.80	88.00	82.60	107.8
	Et_0_ (mm)	0.89	1.25	2.30	2.74	4.43	4.89	5.32	4.38	3.04	1.76	1.16	0.86
	T min (°C)	4.76	5.20	6.73	10.60	14.32	17.25	19.76	19.44	17.12	15.49	10.58	9.02
2006	T max (°C)	10.58	12.03	14.29	19.88	25.42	26.06	30.22	30.38	26.11	23.25	18.16	15.67
	rainfall (mm)	73.60	57.20	44.80	41.20	18.60	22.20	31.20	3.20	97.00	30.00	29.80	79.60
	Et_0_ (mm)	0.88	1.34	2.15	3.31	4.64	5.28	5.75	4.94	3.23	2.27	1.47	0.87

**Table 2:** Parameters from linear regression analysis (b_1_, SE_b_, r^2^, *p* values) between PDWP and SWP for each year (Y), irrigation treatments (I) and soil (S) (entisol and vertisol). T-values and probability values refer to the comparison of slopes (*t*-test) for the two soil types for each irrigation and year combination.

	Year	2005	2006	2005	2006
Soil	Irrigation	Rain-Fed		RDI	
Entisol	b_1_	0.922	1.655	0.952	0.629
	SE_b_	0.11	0.11	0.25	0.09
	r^2^	0.77	0.95	0.44	0.86
	*p*-value	<0.001	<0.001	<0.001	<0.001
Vertisol	b_1_	1.084	1.398	1.264	0.725
	SE_b_	0.15	0.09	0.11	0.12
	r^2^	0.70	0.92	0.87	0.83
	*p*-value	<0.001	<0.001	<0.001	<0.001
Student’s *t*-test	t-value	0.870	1.874	1.150	0.611
	*p*-value	0.39	0.07	0.26	0.55

**Table 3:** Parameters from linear regression analysis (b_1_, SE_b_, r^2^, *p* values) between PDWP and SWP for years (Y) and irrigation treatments (I). Data of the two soils were pooled together.

Year	2005		2006	
Irrigation	Rain-Fed	RDI	Rain-Fed	RDI
b_1_	1.006	1.122	1.498	0.676
SE_b_	0.009	0.127	0.076	0.073
r^2^	0.79	0.66	0.91	0.84
*p* value	<0.001	<0.001	<0.001	0.001

**Table 4:** Tests (Student’s t) for the difference between linear regression slopes.

		*t*-value	*p*-value
rain-fed	2005 *vs.* 2006	6.428	0.000
RDI	2005 *vs.* 2006	3.045	0.003
2005	rain-fed *vs.* RDI	0.911	0.366
2006	rain-fed *vs.* RDI	7.800	0.000

**Table 5:** Effects of soil and irrigation on vegetative and productive parameters of ‘Nero d’Avola’ grapevines grown in two soils (entisol and vertisol) and under rain-fed and regulated deficit irrigation (RDI) treatments. Means ± standard errors and *p*-values from two-way analysis of variance (ANOVA).

	TLA	Cane	Yield	Cluster	Berry
	(cm^2^/shoot)	(g)	(kg/vine)	(g)	(g)
Soil					
Entisol	4233 ± 169	58.0 ± 5.0	2.41 ± 0.14	198 ± 9.0	1.51 ± 0.01
Vertisol	4909 ± 170	79.0 ± 8.0	3.36 ± 0.19	278 ± 25	1.76 ± 0.03
Irrigation					
Rain-fed	4071 ± 244	64.0 ± 6.0	2.68 ± 0.14	220 ± 19	1.48 ± 0.01
RDI	5071 ± 345	73.0 ± 7.0	3.09 ± 0.17	257 ± 14	1.79 ± 0.02
Main factors	*p*-value	*p*-value	*p*-value	*p*-value	*p*-value
Soil (S)	<0.001	<0.001	0.001	0.008	0.012
Irrigation (I)	<0.001	0.065	0.007	0.011	<0.001
Interaction					
S × I	0.207	0.155	0.954	0.749	0.052

**Table 6:** Effects of soil and irrigation on total soluble solids (TTS), titratable acidity (TA), anthocyanin concentration (mg kg^−1^ of grapes) and anthocyanin content (mg/berry) of ‘Nero d’Avola’ grapevines grown in two soils (entisol and vertisol) and under rain-fed and regulated deficit irrigation (RDI) treatments. Means ± standard errors and *p*-values from two-way ANOVA.

	TSS	TA	Anthocyanin	Anthocyanin
	(°Brix)	(g L^−1^)	(mg kg^−1^)	(mg/berry)
Soil				
Entisol	22.70 ± 0.15	6.62 ± 0.07	1293 ± 41	1.92 ± 0.09
Vertisol	22.90 ± 0.17	6.94 ± 0.12	1169 ± 65	2.02 ± 0.07
Irrigation				
Rain-fed	22.85 ± 0.11	7.15 ± 0.09	1343 ± 52	1.97 ± 0.09
RDI	22.75 ± 0.21	6.41 ± 0.10	1119 ± 54	1.97 ± 0.06
Main factors	*p*-value	*p*-value	*p*-value	*p*-value
Soil (S)	0.306	0.045	0.040	0.412
Irrigation (I)	0.451	<0.001	<0.001	0.971
Interaction				
S × I	0.640	0.154	0.197	0.651

## Data Availability

The data presented in this study are available on request from the corresponding author.

## Supplementary Materials

The following are available online at https://www.mdpi.com/article/10.3390/plants10040641/s1, Figure S1: Experimental design, Table S1: Experimental site, irrigation treatment and soil characteristics where the trial was carried out.

## Abbreviations

RDI: regulated deficit irrigation; ANOVA, analysis of variance; TLA, total leaf area; TA, titratable acidity; TSS, total soluble solids, SWP, stem water potential; PDWP, predawn water potential; g_s_, stomatal conduttance; Y, year; I, irrigation treatments; S, soils; PS, pea size; H, harvest.
