# Supplementary material for: Soil and Regulated Deficit Irrigation Affect Growth, Yield and Quality of ‘Nero d’Avola’ Grapes in a Semi-Arid Environment
Source: Plants (Basel). 2021 Mar 28;10(4):641. doi: 10.3390/plants10040641 (PMC8065923; doi:10.3390/plants10040641)

(Barbagallo et al., Supplementary material)

Table S1. Experimental site, irrigation treatment and soil characteristics where the trial was carried out.

Geographical Coordinates	37° 55′ 11,66′′ N; 13° 04′ 10,03′′ E
Altitude	300 m a.s.l.
Cultivar	Nero d'Avola
Rootstock	1103 P
Training system	vertical shoot positioning
Pruning system	single cordon spur pruned
Planting distances	2.40 m x 0.95 m

**Irrigation treatment**		
Regulated deficit irrigation	up to the end of veraison	25% of ET_c
	up to 10 days before harvest	10% of ET_c
Total irrigation volume	2005	42 mm
	2006	28 mm
Irrigation dates (DOY)	2005	173, 191, 204
	2006	206, 234

**Soil**	**Entisol**	**Vertisol**
Depth (cm)	50-60	>100
Sand (%)	26.6	34.0
Loam (%)	32.5	24.6
Clay (%)	40.9	41.3
Coarse fragments	low	low
pH	7.81	7.58
Total salinity (mS/cm)	0.85	1.07
Total $CaCO_3$ (%)	5.4	5.1
Organic matter (%)	0.98	2.03
N content (ppm)	658	966
Exchangeable K_2O	294	402
Exchangeable NaO (ppm)	68	44
Exchangeable CaO (ppm)	3560	3510
Exchangeable MgO (ppm)	419	594

Figure S1. Experimental design

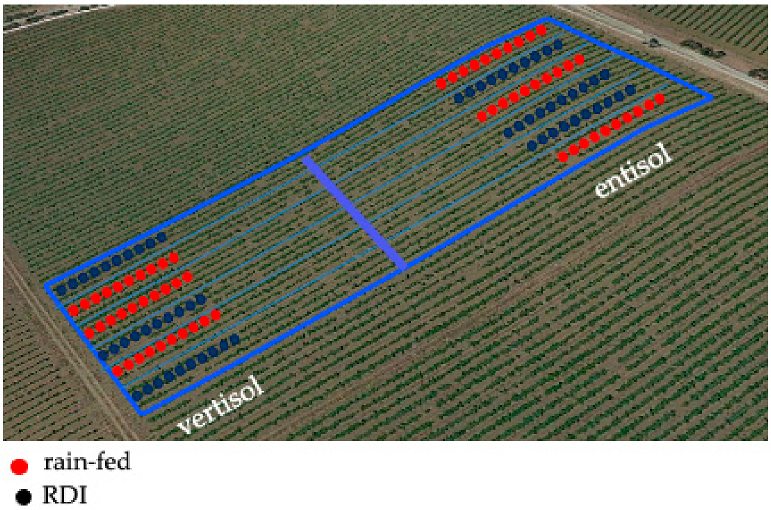

Supplement: Supplementary file 1 [file plants-10-00641-s001.pdf]
